# Dose-Dependent and Lasting Influences of Intranasal Vasopressin on Face Processing in Men

**DOI:** 10.3389/fendo.2017.00220

**Published:** 2017-09-22

**Authors:** Daniel Price, Debra Burris, Anna Cloutier, Carol B. Thompson, James K. Rilling, Richmond R. Thompson

**Affiliations:** ^1^Maine Medical Center, Department of Psychiatry, Portland, ME, United States; ^2^Biostatistics Center, Bloomberg School of Public Health, Johns Hopkins University, Baltimore, MD, United States; ^3^Department of Anthropology, Emory University, Atlanta, GA, United States; ^4^Department of Psychiatry and Behavioral Science, Emory University, Atlanta, GA, United States; ^5^Center for Translational Social Neuroscience, Emory University, Atlanta, GA, United States; ^6^The Center for Social Neuroscience, Atlanta, GA, United States; ^7^Psychology Department and Neuroscience Program, Bowdoin College, Brunswick, ME, United States

**Keywords:** social behavior, V1a receptor, face processing, intranasal, social context

## Abstract

Arginine vasopressin (AVP) and related peptides have diverse effects on social behaviors in vertebrates, sometimes promoting affiliative interactions and sometimes aggressive or antisocial responses. The type of influence, in at least some species, depends on social contexts, including the sex of the individuals in the interaction and/or on the levels of peptide within brain circuits that control the behaviors. To determine if AVP promotes different responses to same- and other-sex faces in men, and if those effects are dose dependent, we measured the effects of two doses of AVP on subjective ratings of male and female faces. We also tested if any influences persist beyond the time of drug delivery. When AVP was administered intranasally on an initial test day, 20 IU was associated with decreased social assessments relative to placebo and 40 IU, and some of the effects persisted beyond the initial drug delivery and appeared to generalize to novel faces on subsequent test days. In single men, those influences were most pronounced, but not exclusive, for male faces, whereas in coupled men they were primarily associated with responses to female faces. Similar influences were not observed if AVP was delivered after placebo on a second test day. In a preliminary analysis, the differences in social assessments observed between men who received 20 and 40 IU, which we suggest primarily reflect lowered social assessments induced by the lower dose, appeared most pronounced in subjects who carry what has been identified as a risk allele for the V1a receptor gene. Together, these results suggest that AVP’s effects on face processing, and possibly other social responses, differ according to dose, depend on relationship status, and may be more prolonged than previously recognized.

## Introduction

Arginine vasopressin (AVP) and related peptides, including its ancestral, non-mammalian homolog, arginine vasotocin (AVT), act as central neuromodulators across vertebrates that regulate, among other functions, social behavior [reviewed in Ref. (1–3)]. Many of these effects are associated with influences in a conserved network of nuclei within the brain, the Social Brain Network (SBN), that are reciprocally connected and regulate a variety of social behaviors across vertebrates (4–6). The production of AVT/AVP within several of these nodes has been highly conserved, though the projections from these nodes and the distributions of peptide receptors are highly variable across species, including numerous target sites outside of the traditional SBN. This variation likely accounts for the diversity of behavioral effects these peptides have across species [reviewed in Ref. (7)].

In addition to their species-specific influences, AVT/AVP’s behavioral effects can differ between the sexes, between individuals of the same sex that display alternative phenotypes, and as a function of complex dose-dependencies. For example, in tropical damselfish, AVT’s ability to stimulate aggression in males follows an inverted U function, with mid-range doses being most effective (8). This suggest that higher doses do not simply produce maximal behavioral output upon receptor saturation, but may have influences that counteract those of lower doses or induce alternative behavioral responses, perhaps by activating different patterns of receptors across the SBN. Sex/phenotype-specific influences include cases in which behavioral patterns only exhibited by one sex are affected (most often male-typical behaviors, as in the damselfish example above); cases in which the peptide induces opposite effects in the sexes (9–12), and even cases in which the peptide has different effects in individuals of the same sex that adopt alternative mating strategies (13).

Arginine vasotocin/arginine vasopressin can also produce context-dependent effects, as has been most elegantly demonstrated in birds. In the territorial estrildid violet-eared waxbill, exogenous administration of a V1a receptor antagonist reduced aggression related to mate competition in males, but did not affect resident-intruder aggression (14), and in zebra finches the antagonist reduced aggression during mate competition but increased aggression following colony establishment (15). Some context-dependent effects are a simple function of the sex of the stimulus present; knockdown of AVT production in the paraventricular nucleus of zebra finches enhances aggression toward females in males, but not toward other males (11). Similarly, AVP promotes affiliative responses toward females in male prairie voles, but promotes aggressive responses toward other males (16). Most of these context/stimulus-dependent effects likely depend on the activation of different AVT/AVP circuits that produce unique behaviors in response to particular social stimuli. For example, AVP’s ability to stimulate aggression in male prairie voles depends on actions within the hypothalamus (17), whereas its ability to promote affiliative response toward females depends on actions within the ventral pallidum and septum (18, 19).

Although we know a great deal about the acute effects of AVT/AVP, we know very little about whether or not there might be long-term consequences associated with those acute effects. Work with oxytocin (OT) in mammals and AVT in birds demonstrates that nonapeptides can have long-term effects on the brain and social behavior during early developmental windows (20, 21) [reviewed in Ref. (22)], though perhaps through mechanisms that are only operative during critical periods. In adults, AVP affects social recognition/memory, though such effects are typically evident only 2–24 h after AVP manipulations (23, 24). Even exogenous AVP’s effects on affiliative responses related to pair bonding in male prairie voles have only been examined immediately after manipulations of AVP that are concurrent with social interactions with females (16, 18). Intriguingly, though, recent work indicates that mating, which induces the AVP release necessary for pair bonding in this species, triggers epigenetic changes that enhance affiliative behavior (25, 26). Whether those epigenetic changes depend upon mating-induced AVP release, or how long their influences upon behavior persist, remain to be determined.

Examination of the role AVP plays in human social behaviors a has thus far been much less extensive than in other animals. However, there is emerging evidence of a diversity of effects, some of which are sex- and context specific. As in male prairie voles, there is some data suggesting a role in pair bonding; allelic variation within the RS3 domain of the promoter for the V1a receptor is associated with pair bond strength in men (27). Studies in which AVP is intranasally delivered, which elevates peptide levels in the brain (28), have more directly implicated AVP in social regulation. AVP selectively draws attention to sexual content in language (29) and increases empathic concern in both male and female subjects who had previously received high levels of warmth from their fathers (30). Intranasal AVP also facilitates cooperation in complex social decision tasks, effects that are dependent on sex, contexts of the task, and the personality of the individual (31–33).

The above-mentioned effects likely promote social engagement, consistent with the ability of nonapeptides to promote affiliative/courtship-related responses in other animals and/or in some contexts. However, AVP/AVT can also enhance aggression and social withdrawal. Consistent with the possibility that AVP might likewise have antisocial effects in humans, levels of AVP correlate positively with life histories of aggression in men (34). Also, intranasal AVP delivery not only affects the processing of positive emotions in faces but also negative ones (35, 36), and it decreases how friendly men rate the faces of unfamiliar men while enhancing facial expressions consistent with negative, and possibly even threat-related, responses (37, 38). However, we do not yet know if the negative effects in men depend on context, in this case of the stimulus sex. AVP might, as in male prairie voles, promote antisocial responses toward other males but facilitate affiliative responses toward potential mates. We also do not yet know if AVP might produce dose-dependent influences on the ratings of faces, or if any of its effects on subjective face ratings could have long-lasting consequences.

To address those questions, we compared the effects of two doses of AVP commonly used in intranasal studies, 20 and 40 IU, on subjective ratings of same- and other-sex faces in human males 50 min after drug or placebo delivery and again multiple days later (between 2 and 20 days after drug delivery). Because differences in tendencies to form emotional attachments are related to variation in the RS3 domain of the V1a receptor prompter, we also ran models that included variation in RS3 alleles. We had five primary predictions. First, that 20 IU would, as in our previous study, decrease ratings of same-sex faces, but possibly increase ratings of female faces if, as discussed above, mechanisms similar to those in prairie voles are operative in humans. Second, if AVP does enhance positive responses toward females, then the effects might persist on follow-up tests when no drug is delivered. Third, that influences of AVP on responses to male and female faces might differ in single men and those in relationships in light of findings that social experience, including pairing, can induce changes in AVP circuitry in other animals. Fourth, that any negative effects of AVP would be greater in men who carry V1a RS3 risk alleles, and any positive responses smaller in those individuals. Fifth, that the higher dose would produce similar, but more pronounced effects than the lower dose if the dose–response function is linear, but divergent effects if it is not.

## Materials and Methods

### Subjects

Male subjects between the ages of 18 and 30 were recruited through advertisements in Craig’s list in Portland, Maine, the local gym, newspaper and community college, Maine Medical Center’s electronic newsletter, as well as through referrals. Of those who responded, 94 passed our initial screenings and consented to participate. Seven subjects withdrew following Treatment Day 1 and one after Treatment Day 2 for various, non-study-related reasons. Of the 86 subjects who completed all 3 days, 2 were African-American, 3 were Asian, and 2 were Hispanic. The remaining 79 subjects were Caucasian. Data for those who only completed day 1 were used in between-subjects comparisons on that day, and data for subjects who only completed the first 2 days were used in within-subjects comparisons across those days.

All subjects were initially interviewed by phone for a pre-screen to exclude subjects that were prescribed serotonin reuptake inhibitors or had cardiovascular or neurological conditions, cancer, asthma, facial Botox, or substance abuse issues. Those who passed this pre-screen then came for an in-person screen at Maine Medical Center. At that time, verbal and written informed consent were obtained from each subject. Subjects were provided a copy of the informed consent document. Following consent, subjects were assigned an ID number and asked to provide a urine sample for drug testing. Demographic information, sexual orientation, and relationship status were recorded. Subjects were then given a physical exam, including EKG. Exclusion criteria were hypertension [systolic blood pressure (BP) >140 and/or diastolic BP >90], hypotension (systolic BP <90 and/or diastolic BP <50), temperature >100, and/or a positive drug screen. All subjects were examined by a board-certified psychiatrist and screened in a semi-structured interview for ongoing Axis I psychiatric or substance abuse. Any active Axis I disorder requiring ongoing treatment led to exclusion from participating in the study, as did any acute psychiatric symptoms (e.g., delusions, hallucinations, paranoia, mania, depression, obsessions, compulsions, or severe anxiety) evident at the time of the interview. Initially, subjects were paid $300 if they completed all visits, prorated to $50 at screening, $100 at treatment day 1 and 2 and $50 at non-treatment day if they did not complete all three test days in addition to the initial screening. However, due to difficulties recruiting subjects, we increased the amount paid to $500, prorated to $100 at screening, $150 on treatment days 1 and 2 and $100 on non-treatment day if they did not complete all three test days.

The study was approved by the Bowdoin College and Maine Medical Institutional Review Boards and by the U.S. Food and Drug Administration. None of the subjects developed any major side effects in response to AVP, including anaphylaxis.

### Drugs

Sterile, lyophilized AVP was purchased from PolyPeptide Laboratories (Sweden). Drug was dissolved in sterile saline by the pharmacy at Maine Medical Center in two doses; 20 IU/0.5 ml and 40 IU/0.5 ml, drawn into 1 cc syringes, then immediately frozen and stored at −80°C until use. Placebo vials contained the same volume of sterile saline and were likewise stored at −80°C. Drugs were sent out for tests of efficacy every 6 months to Eagle Analytical Services. All tests showed that both doses retained their full efficacy throughout the test period (remained within 10% of appropriate international units). No drug was used after more than 12 months storage. The pharmacy also created randomization tables that assigned each subject to one of the two doses, to either getting drug or placebo on day 1, and to the stimulus sets that would be seen on each test day (see further explanation below). All study personnel were blind to whether the subject received placebo or drug on a given day and to what dose the subject would get on the drug day.

### Stimuli

Photographs were taken of female and male models by a professional photographer. We chose Caucasian models because, given racial demographics in Maine, we anticipated the overwhelming majority of subjects would be Caucasian. For reasons related to our hypothesis that AVP would affect responses to specific individuals, we only wanted to show a single male and a single female after AVP and after placebo, and we did not include multiple models that differed by race in hopes of minimizing variation related to in-group/out-group influences. We used only neutral emotional expressions to determine if AVP can bias individuals to respond to ambiguous social stimuli more negatively or positively. Images from multiple models were initially piloted with Bowdoin undergraduates to select the two female and the two male models who appeared most similar in terms of basic features like hair color and whose pictures were rated most similarly on responses measured during the study. Measurements included Approachability (from −3, which indicated the face was threatening and not approachable, to 3 for faces subjects felt were friendly and very approachable), Willingness to Initiate Conversation with the person (Initiate; from −3, not likely at all, to 3, very likely), and Attractiveness (−3, very unattractive, to 3, very attractive). These responses were chosen to try to dissociate responses related to social perception (Approachability), social motivation (Initiate), and sexual/romantic potential (Attractiveness).

Five stimulus sets were created, four for the first two test days, and one for the third, final day (FD) of testing. Each of the four sets for days 1 and 2 contained 18 images, 9 of one female and 9 of one male, each taken in different lighting and with different postures to create some variability, in pseudorandomized order so the same individual was never presented more than twice in a row. The sets, therefore, consisted of the four possible combinations of individual male and female faces that could be seen together. The final day stimulus set consisted of 36 images and included the same 9 images of each of the four models seen previously on the first two test days, in a pseudorandomized order that ensured the same face was not presented more than twice in a row.

### Experimental Design

We employed a within-subjects design in which each subject received placebo on one test day and one of the two doses of AVP on the other, in counterbalanced order. The stimulus sets seen by each subject on placebo and drug days were assigned randomly. Thus, subjects saw one of the female models and one of the male models after placebo, and the other female and the other male after drug. On the final day, when no drug was administered, subjects saw the stimulus set that included all of the faces previously seen.

### Procedure

Subjects were met at the study site by the research nurse who conducted the procedures. Adverse life events since screening, concomitant medications, fluid, and caffeine intake were reviewed. If all treatment criteria were met, the site investigator provided a written prescription for study drug to the pharmacy. The pharmacy then randomized the subject and delivered study drug syringe, with a MAD300 Nasal Atomization Device attached. Once the subject was settled and comfortable, the research nurse proceeded to prepare the subject for facial electromyographic, skin conductance, and heart rate recordings, but that data will not be presented in this paper. Subjects were attached to an automatic BP, pulse, and temperature monitor. Baseline (pre-study drug) readings were collected. Subjects were then asked to self-administer the study drug in a single dose to one nostril (20 IU/0.5 ml, 40 IU/0.5 ml, placebo-sterile saline). Subjects then viewed a neutral 30 min DVD, Blue Planet: Seas of Life. Serial BP, pulse, and temperature measurements were taken at baseline (pre-study drug administration), and again at 5, 20, 30, and 60 min post study drug administration, though only BP from baseline, 20 and 60 min were analyzed statistically.

Image presentation began 50–60 min after drug administration (Stroop Software; Coulbourn Instruments). Each face was presented on a computer screen 36 inches in front of the subject for 8 s, 20–30 s apart. The interval between images varied randomly between 20 and 30 s to keep subjects from anticipating exactly when each image would be presented within that window. Subjects observed a blank screen between images. Immediately after each image disappeared, the technician running the session asked the subject to say how approachable the face was, on the scale discussed above, how likely the subject would be to initiate conversation with the person whose face was shown, and how attractive the subject thought the face *was*. All verbal responses were recorded by the research nurse. Immediately following image viewing, 2 EDTA (ethylenediaminetetraacetic acid—a standard additive chelating agent that binds calcium and other metals, thus preventing coagulation of specimens) tubes of blood were collected *via* peripheral phlebotomy.

These procedures were repeated for treatment day 2, which occurred 2–7 days after day 1. Subjects reported back for the final test day no sooner than 2 days since the day 2 trial and no more than 21 days from the initial screening. Thus, the final test day occurred within 2–20 days of AVP administration. All of the procedures were repeated on the final test day, but subjects did not self-administer placebo or drug. They observed and responded to the stimulus set containing 36 images, 9 each of all the faces previously observed. Two female nurse/technicians collected data, but the same person collected all data across days from each individual.

### Microsatellite Genotyping

Genotypes for the RS3 microsatellite at *AVPR1A* were determined according to the method described in Kim et al. (39). Briefly, a PCR with fluorophore-labeled primers was performed using the following conditions: 1XBuffer (Applied Biosystems), 2.5 mM MgCl_2_, 0.5 mM forward-RS3 primer (6-FAM-TCCTGTAGAGATGTAAGTGC); 0.5 mM reverse-RS3 primer (gtttcttTCTGGAAGAGACTTAGATGG), 0.08 mM dNTP, 0.06 U Amplitaq Gold (Applied Biosystems). 5 µl of this assay mix was added to a 384 plate containing 10 ng of dried DNA. Amplification cycles were executed in a 9700 Gene Amp PCR System (Applied Biosystems) at the following conditions: 95°C for 5 min; 94°C for 30 s, 55°C for 30 s, 72°C for 1 min (35 cycles), and 72°C for 10 min. PCR products were then subjected to electrophoresis and laser detection of product on an ABI 3100 System, and data analyzed using *Gene Mapper Software (Applied Biosystems)*. Each electropherogram was checked visually to confirm calls assigned by the Software, and ambiguous calls were either resolved by consensus of two experienced readers, or discarded and repeated. Quality control included the analysis of positive and negative controls, duplicate samples and Hardy Weinberg Equilibrium tests.

### Statistical Analysis

Statistical analyses were performed by the Biostatistics Center at Johns Hopkins University. Data were reviewed, and sequences of facial assessments were exluded on the drug day for two subjects for whom the nursing log indicated that substantial amounts of drug had been lost during delivery due to problems the subjects had with the self-administration. For BP, the percent change from baseline 20 and 60 min after placebo and drug administration were used as the outcome measure, with initial baseline BP as a covariate. For behavioral scores, medians of scores within each stimulus sex under each condition were calculated where there were at least five scores available (equipment/software problems caused the Stroop program to stop running before the trial was complete in 1 case). Medians were considered the outcome measure for all analyses.

General linear models were performed on all analyses using IBM SPSS Statistics v 24 (IBM, Inc., Armonk, NY, USA). Relationship Status was a factor in our initial models of behavioral responses because we predicted AVP might differentially affect responses in single and coupled men. That prediction was supported in our initial models, so subsequent models were stratified by Relationship Status.

For day 1 analyses, Dose (0, 20, 40 IU) was a between-subjects factor; repeated measure was Stimulus Sex. For analyses across test days and on the final day, factors in the model were Drug Order and Dose; repeated measures were Stimulus Sex and Drug (AVP, Placebo). For those models, significant (*p* < 0.05) main effects and significant or marginal (0.1 < *p* < 0.05) interactions were evaluated, as were pairwise comparisons that tested specific predictions. Marginal interactions were interrogated to evaluate potentially important relationships between the factors/repeated measures and outcomes, recognizing that the power to detect a significant interaction was likely not adequate given the sample size (40, 41). For the highest order interactions, pairwise comparisons of Dose and Drug were made at each level of Stimulus Sex and other factors or repeated measures within the interaction using the Sidak adjustment for multiple comparisons (42). *p*-Values noted are adjusted. We do not report significant interactions in cases where pairwise comparisons failed to detect significant differences between treatment condition (drug vs placebo) or Dose (20 vs 40 IU).

To control for individual differences in responses potentially associated with V1a allelic variation, we included whether subjects had 0, 1, or 2 copies of the 335 allele, which corresponds to the 334 risk allele identified by others (39, 43) with the primers we used, in our models. We also included whether subjects had 0, 1, or 2 long alleles (≥335). To determine if variation in RS3 influences responsiveness to AVP, we ran models for men who received 20 or 40 IU on day 1, which our initial analyses indicated was the only time when drug administration produced effects, that included whether or not subjects had at least one copy of the 335 allele as a factor.

## Results

### Blood Pressure

There were no significant differences in mean percent change in systolic or diastolic BP 20 or 60 min after administration across subjects who received placebo, 20 or 40 IU on day 1 of testing. Across test days, there were not main effects for Dose or interactions with Drug Order for either dose for mean percent changes in systolic or diastolic pressure from baseline 20 min after administration, nor for percent changes in diastolic pressure 60 min after administration. The mean percent change in systolic pressure, however, was significantly lower 60 min after the administration of 40 IU than 60 min after the administration of placebo (Drug main effect, *p* = 0.01; mean ± SEM: placebo, −0.3% ± 1.1, 40 IU; −2.0% ± 1.4).

### Behavior

We ran nine models that included Relationship Status as a factor, three for responses on the first day across subjects who got placebo, 20 or 40 IU (1 model for each variable), three for responses across the first two test days when subjects received placebo on one day and 20 or 40 IU on the other, and three for responses on the final day. There were significant interactions between Dose, Stimulus Sex, and Relationship Status for seven of the nine models (*p* < 0.01 for all but Approachability on day 1 and Willingness to Initiate Conversation across days 1 and 2), supporting the stratification by Relationship Status. We, therefore, report the results from the same nine models stratified by Relationship Status.

### Day 1 Between-Subjects Comparisons Stratified by Relationship Status

#### Approachability

**Figure 1:**
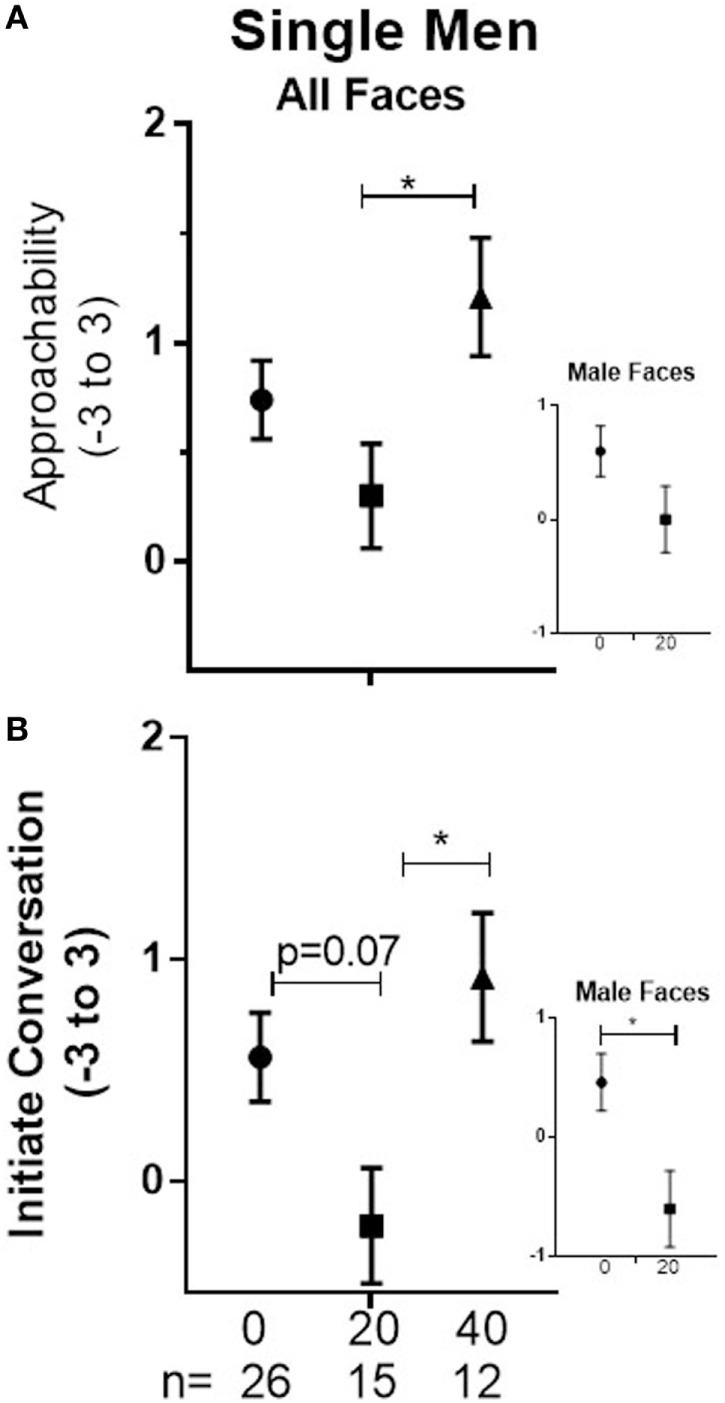
Men ± SEM of Approachability **(A)** and Initiate ratings **(B)** of faces (averaged across male and female) on day 1 in single men who received placebo (0), 20, or 40 on that day. Inserts show planned, focused comparison between placebo and 20 IU for responses to male faces.

In single men, there was a significant main effect of Dose (*p* = 0.047) associated with significantly lower responses in men given 20 IU than in men given 40 IU (−0.91, 95%CI: −1.79 to −0.03, *p* = 0.042; see Figure 1A). We cannot resolve whether this indicates that 20 IU decreased responses, 40 IU increased them, or both. Because we previously observed that 20 IU decreases ratings of male faces relative to placebo, we did examine the pairwise comparison of placebo vs 20 IU for male faces, which was not significant (see Figure 1A). There were no differences between doses for coupled men.

#### Initiate

In single men, there was a significant main effect of Dose (*p* = 0.02) that was associated with a marginally lower mean in subjects given 20 IU than placebo (0.76, 95%CI: −1.6 to 0.05, *p* = 0.07) and a significantly lower mean in subjects given 20 than 40 IU (−1.12, 95%CI: −2.09 to −0.15, *p* = 0.02; see Figure 1B). Because we previously observed that 20 IU decreases ratings of male faces relative to placebo, we did examine the pairwise comparison of placebo vs 20 IU for male faces; mean responses were significantly lower in men given 20 IU than in men given placebo (−1.06, 95%CI: −2.05 to −0.08, *p* = 0.03; see Figure 1B). There were no differences between doses for coupled men.

#### Attractiveness

**Figure 2:**
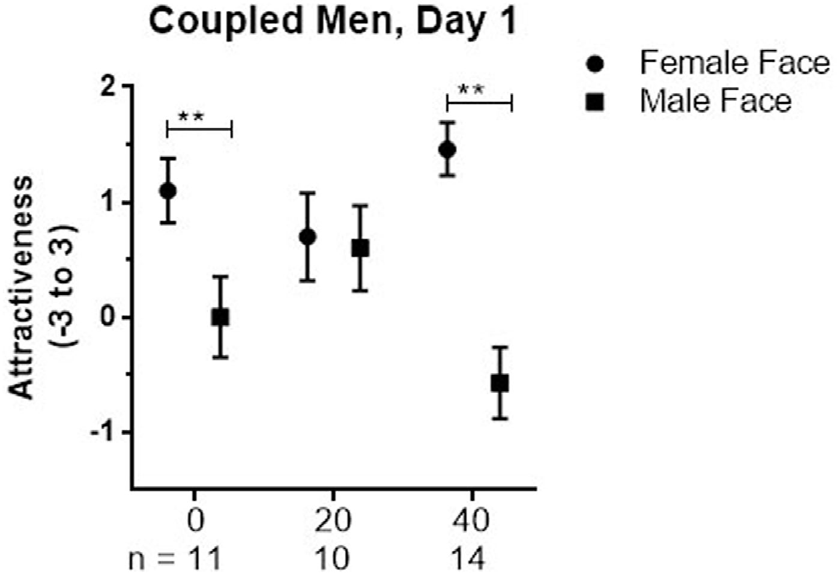
Mean ± SEM of Attractiveness ratings of female and male faces on day 1 in coupled men who received placebo (0), 20, or 40 IU on that day.

There were no significant main effects or interactions for single men, nor was the planned comparison of responses to male faces between men given 20 IU and placebo significant. In coupled men, the Dose × Stimulus Sex interaction was significant (*p* = 0.003). For male faces, the mean response after 20 IU was marginally higher than after 40 IU (1.17, 95%CI: −0.05 to 2.39, *p* = 0.06). The significant interaction was largely due to differences in how coupled men processed female faces relative to male faces; those given placebo and 40 IU rated female faces significantly higher than male faces (placebo: 1.09; 95%Cl: 0.31 to 1.87, *p* = 0.008; 40 IU: 2, 95%Cl: 1.31 to 2.69, *p* < 0.001; see Figure 2), whereas those given 20 IU did not. This pattern could reflect a tendency for men given 20 IU to rate female faces lower, male faces higher, or both.

### Days 1 and 2 Within- and Between-Subjects Comparisons Stratified by Relationship Status

#### Approachability

##### Single Men

**Figure 3:**
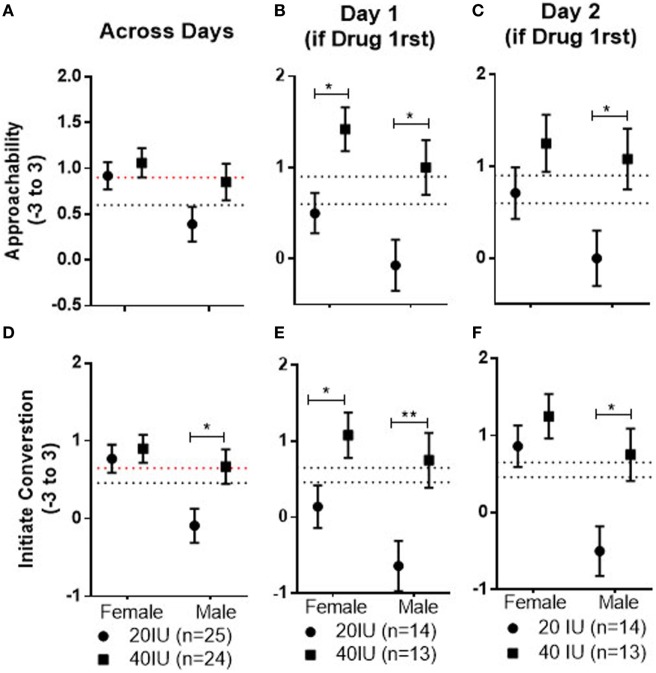
Mean ± SEM of Approachability (top) and Initiate (bottom) ratings to female and male faces, averaged across both test days, in men who received 20 or 40 IU on either day **(A,D)**, to female and male faces observed after drug on day 1 in men who received 20 or 40 IU **(B,E)**, and to female and male faces observed after placebo on day 2 in men who received 20 or 40 IU on day 1 **(C,F)**. The top dotted line shows the mean response to female faces on day 1 in men who received placebo on that day, the bottom dotted line the mean response to male faces on day 1 in men who received placebo on that day.

There was no drug effect for either the 20 or 40 IU dose compared with placebo in the within-subject comparison. However, there was evidence for dose differences that persisted over time, particularly if AVP was administered on day 1. There was a significant Dose × Drug Order interaction (*p* = 0.01). The mean for responses to the faces across days and Stimulus Sex was significantly lower in men given 20 IU than in men given 40 IU if drug was given on day 1 (−0.90, 95%CI −1.53 to −0.28; *p* = 0.006; not shown, but see further analysis below, as summarized in Figure 3). Additionally, the mean response to faces across days and Stimulus Sex was significantly lower in men given 20 IU on day 1 than in men given 20 IU on day 2 (−0.74; 95%CI: −1.38 to −0.1; *p* = 0.03; not shown). This pattern is consistent with the possibility that the dose difference is, at least in part, associated with decreased responses induced by 20 IU if given on day 1. Because responses across days differed as a function of the dose given on day 1, we wanted to dissociate and test for acute and carry-over effects through which AVP, if given on day 1, may have affected responses across the days. Therefore, we examined pairwise comparisons between men given 20 and 40 IU on day 1 for faces seen after drug on day 1 and after placebo on day 2, even though the Drug × Dose × Stimulus Sex × Drug Order interaction was not significant. Consistent with acute differences on day 1, the mean for responses to female and male faces were both significantly lower after 20 IU than after 40 IU on day 1 (female faces: −0.92, 95%CI: −1.57 to −0.26, *p* = 0.007; male faces: −1.07, 95%CI: −1.9 to −0.18, *p* = 0.01; see Figure 3B). Consistent with carryover effects that generalized to new male faces, the mean response to male faces seen after placebo on day 2 was significantly lower for men that had been given 20 than 40 IU on day 1 (−1.08, 95%CI: −1.98 to −0.18, *p* = 0.02; Figure 3C). In contrast, the mean for female faces seen after placebo on day 2 did not differ between subjects that had been given 20 and 40 IU on day 1 (−0.54, 95%CI: −1.37 to 0.32, *p* = 0.2).

##### Coupled Men

**Figure 4:**
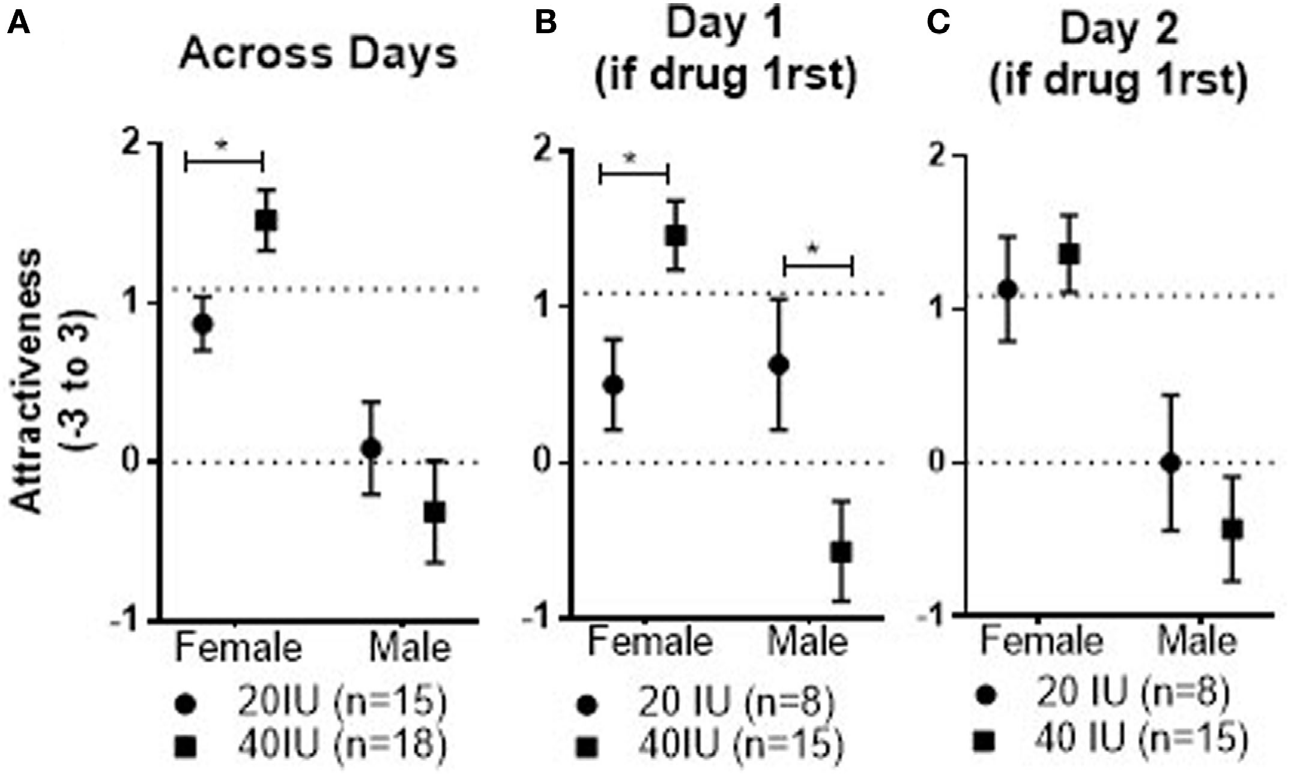
Mean ± SEM of Attractiveness ratings of male and female faces across days in coupled men who received 20 or 40 IU on either day **(A)**, as well as responses on day 1 **(B)** and day 2 **(C)** in coupled men who received 20 or 40 IU on day 1. The top dotted line shows the mean response to female faces on day 1 in coupled men who received placebo on that day, the bottom dotted line the mean response to male faces in men who received placebo on day 1.

There was no drug effect for either the 20 or 40 IU dose compared with placebo in the within-subject comparison. However, here was a significant Dose × Stimulus Sex interaction (*p* = 0.01). Regarding female faces, the mean across test days was significantly lower in coupled men given 20 IU than in coupled men given 40 IU (−0.72, 95%CI: −1.23 to −0.16, *p* = 0.01; data not shown, but see Figure 4 for a similar pattern for Attractiveness). We cannot resolve whether that reflects lowered responses in men given 20 IU or higher responses in men given 40 IU. The Dose × Stimulus Sex interaction was qualified by a marginal Dose × Stimulus Sex × Drug Order interaction (*p* = 0.06). If placebo was given first, the mean response to female faces across days was significantly lower in coupled men given 20 IU on day 2 than in those given 40 IU (−1.09, 95%CI: −2.0 to −0.17, *p* = 0.02). This pattern is difficult to interpret, but it suggests that of the coupled men given placebo first, there may have been initial differences in how those who subsequently received 20 and 40 IU on day 2-rated female faces. The possibility for such sampling error was high for that comparison because only six coupled men received placebo on day 1 and 20 IU on day 2, and only four received placebo on day 1 and 40 IU on day 2. Thus, it is important to exercise caution when interpreting order effects associated with placebo administered on day 1 and AVP on day 2 in coupled men.

#### Willingness to Initiate Conversation Stratified by Relationship Status

##### Single Men

There was no drug effect for either the 20 or 40 IU dose compared with placebo in the within-subject comparison. However, there was evidence for dose differences that persisted over time, particularly if AVP was administered first. There was a significant Dose × Stimulus Sex interaction (*p* = 0.008); the mean response to male faces seen across both days was significantly lower in men given 20 IU than in those given 40 IU (−0.76, 95%CI: −1.39 to −0.13, *p* = 0.02; see Figure 3D). There was also a significant Dose × Drug Order interaction (*p* = 0.04); the mean response to all faces across both test days was significantly lower for subjects given 20 IU on day 1 than for subjects given 40 IU on day 1 (−0.99, 95%CI: −1.72 to −0.27, *p* = 0.008; not shown, but see further analysis below, as summarized in Figure 3). Additionally, the mean response, across days and sexes, was significantly lower in men given 20 IU on day 1 than in men given 20 IU on day 2 (−0.75, 95%CI: −1.49 to −0.01, *p* = 0.046; not shown), again suggesting the dose differences are, at least in part, associated with decreased responses induced by 20 IU on day 1. Because responses across days differed as a function of the dose given on day 1, we wanted to dissociate and test for acute and carryover effects. We, therefore, examined pairwise comparisons between subjects given 20 and 40 IU on day 1 for faces seen after drug on day 1 and after placebo on day 2, even though the Drug × Dose × Stimulus Sex × Drug Order interaction was not significant. The mean responses to female and male faces on day 1 were significantly lower for subjects given 20 IU than for subjects given 40 IU (female faces: −0.97, 95%CI; −1.77 to −0.11, *p* = 0.03; male faces: −1.39, 95%CI: −2.37 to −0.42, *p* = 0.006; see Figure 3E), consistent with predicted acute differences. Additionally, the mean response to male faces seen after placebo on day 2 was significantly lower for men that had been given 20 than 40 IU on day 1(−1.25, 95%CI: −2.19 to −0.31, *p* = 0.01; see Figure 3F), consistent with carry-over effects that generalized to the new male faces. In contrast, the mean for female faces seen after placebo on day 2 did not differ between subjects that had been given 20 and 40 IU on day 1 (−0.39, 95%CI: −1.18 to 0.4, *p* = 0.32).

##### Coupled Men

No significant main effects or interactions were detected for which follow-up, pairwise comparisons revealed significant differences between treatment conditions (AVP vs placebo) or doses.

#### Attractiveness

##### Single Men

No significant main effects or interactions were detected for which follow-up, pairwise comparisons revealed significant differences between treatment conditions (AVP vs placebo) or doses.

##### Coupled Men

There was no drug effect for either the 20 or 40 IU dose compared with placebo in the within-subject comparison. However, there was a significant Dose × Stimulus Sex interaction (*p* = 0.03); the mean response to female faces observed across test days was significantly lower in men given 20 IU than in those given 40 IU (−0.65, 95%CI: −1.17 to −0.13, *p* = 0.02; see Figure 4A). There was also a significant Drug × Dose × Stimulus Sex × Drug Order interaction (*p* = 0.04). For female faces, the mean for responses to faces observed after drug on day 1 was significantly lower in men given 20 IU than in men given 40 IU (−0.96, 95%CI: −1.7 to 0.23, *p* = 0.01; see Figure 4B). We cannot resolve whether that reflects lower responses in men givne 20 IU or higher responses in men given 40 IU. In contrast, the mean response to male faces seen after 20 IU on day 1 was significantly higher than the mean response after 40 IU on day 1 (1.2, 95%CI: 0.11 to 2.28, *p* = 0.03; see Figure 4B). Unlike what we observed in single men, there were not any carry-over effects of the doses in coupled men, as neither the mean responses to the female nor the male faces seen after placebo on day 2 were significantly different in men who had received 20 and 40 IU on day 1 (see Figure 4C).

### Final Day Comparisons Stratified by Relationship Status

#### Approachability

##### Single Men

**Figure 5:**
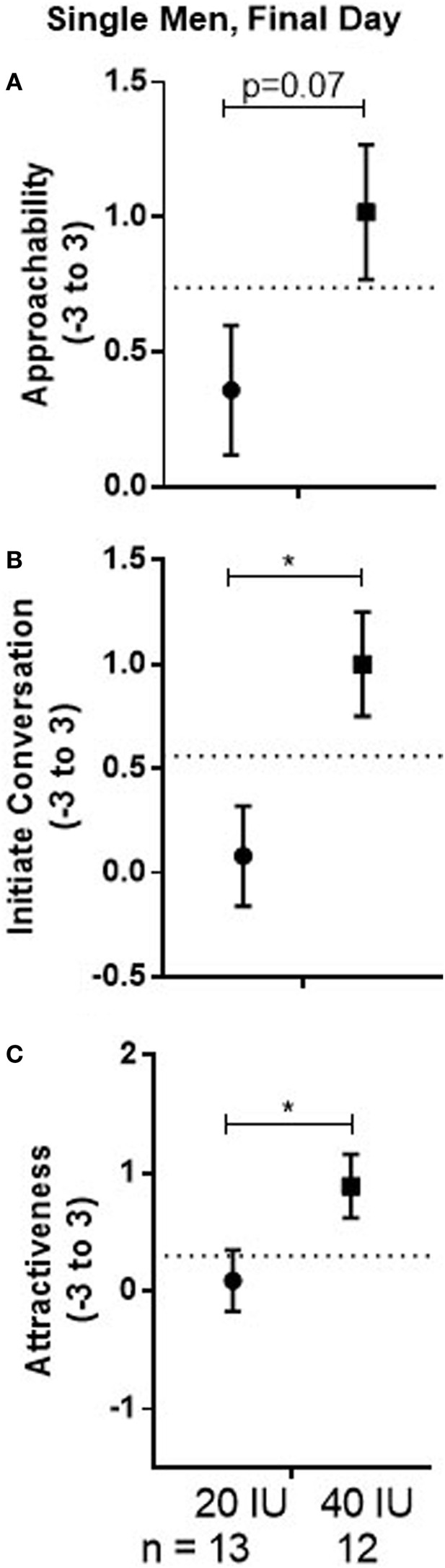
Mean ± SEM of Approachability **(A)**, Initiate **(B)**, and Attractiveness **(C)** ratings, averaged across sex, on the final day of testing when no drug was given in men who received 20 or 40 IU on day 1. The dotted line shows mean response to all faces on day 1 in men who received placebo on that day.

There was no drug effect for either the 20 or 40 IU dose compared with placebo in the within-subject comparison. However, there was evidence for dose differences that persisted over time, particularly if AVP had been administered first, as indicated by a marginal Dose × Drug Order interaction (*p* = 0.05). The mean for responses to all faces previously seen across test days was marginally lower in men given 20 IU on day 1 than in men given 40 IU on day 1 (−0.67, 95%CI: −1.37 to 0.04, *p* = 0.07; see Figure 5A). Additionally, the mean response in men given 20 IU was significantly lower if they had received drug on day 1 than on day 2 (−0.82, 95%CI −1.56 to −0.08, *p* = 0.03; not shown). Together, these results suggest lasting influences of AVP that appear, at least in part, associated with decreased responses induced by 20 IU on the first test day. Consistent with that possibility, responses in men given 20 IU on day 1 remained below the “baseline” responses on day 1 for men given placebo on that day (see dotted lines in Figure 5). In our parallel fMRI study, Approachability ratings of male faces increased across days in single men given placebo or 40 IU on day 1 as a function of experience seeing the faces or simply going through the task (44), which did not happen in single men given 20 IU in the current study.

##### Coupled Men

There was no drug effect for either the 20 or 40 IU dose compared with placebo in the within-subject comparison. However, there was a significant Dose × Stimulus Sex interaction (*p* = 0.001), suggestive of dose differences that extended beyond the time of drug delivery, though not exclusively associated with delivery on day 1. Independent of drug order, the mean response was marginally lower for female faces previously observed across both trials in men given 20 IU than in men given 40 IU (−0.68, 95%CI: −1.36 to 0.1, *p* = 0.05; not shown). Again, we cannot determine if that dose difference is associated with lower responses in men given 20 IU, higher responses in men given 40 IU, or both. There was also a significant Drug × Dose × Stimulus Sex × Drug Order interaction (*p* = 0.01). For female faces, the mean for responses to the faces previously seen after placebo on day 1 was significantly lower in men given 20 IU on day 2 than in men given 40 IU on day 2 (−1.58, 95%CI: −2.94 to −0.23, *p* = 0.02). However, the small number of coupled men who received placebo on day 1 and drug on day 2, as already discussed, make it necessary to exercise caution in interpreting that difference, which may be associated with starting differences between coupled men who received placebo on day 1 and 20 or 40 IU on day 2.

#### Willingness to Initiate Conversation

##### Single Men

There was no drug effect for either the 20 or 40 IU dose compared with placebo in the within-subject comparison. However, there was evidence for dose differences that persisted over time, particularly if AVP was administered on day 1. There was a significant Dose × Drug Order interaction (*p* = 0.03); independent of Stimulus Sex, the mean for responses to all the faces previously observed was significantly lower in men given 20 IU on day 1 than in men given 40 IU on day 1 (−0.92, 95%CI: −1.63 to −0.22, *p* = 0.01; see Figure 5B). Additionally, for subjects given 20 IU, the mean response was significantly lower if the drug had been given on day 1 than if it had been given on day 2 (−0.8, 95%CI: −1.54 to −0.05, *p* = 0.03; not shown).

##### Coupled Men

There was a significant Drug × Dose × Stimulus Sex × Drug Order interaction (*p* = 0.04). The mean for responses to faces previously seen after placebo on day 1 was marginally lower in men given 20 IU on day 2 than in men given 40 IU on day 2 (−1.08, 95%CI: −2.22 to 0.06, *p* = 0.06). However, for reasons discussed above related to small sample sizes and potential starting differences in responsiveness to faces in coupled men who received placebo first and drug second, these differences should be interpreted cautiously.

#### Attractiveness

##### Single Men

There was no drug effect for either the 20 or 40 IU dose compared with placebo in the within-subject comparison. However, there was evidence for dose differences that persisted over time, particularly if AVP was administered on day 1. There was a marginal Dose × Drug Order interaction (*p* = 0.09); the mean response to all faces previously observed on both test days was significantly lower in men who received 20 IU on day 1 than in men who received 40 IU on day 1 (−0.8, 95%CI: −1.55 to −0.05, *p* = 0.04; see Figure 5C). There was also a significant Dose × Stimulus Sex interaction (*p* = 0.03), though pairwise comparisons only detected a marginally lower mean for responses to male faces in men given 20 IU than in men given 40 IU (−0.71, 95%CI: −1.50 to 0.81, *p* = 0.08).

##### Coupled Men

There was a significant Dose × Stimulus Sex interaction (*p* = 0.04); the mean response to female faces was marginally lower in subjects given 20 than 40 IU (−0.53, 95%CI: −1.17 to 0.1, *p* = 0.097). There was also a significant Drug × Dose × Stimulus Sex × Drug Order interaction (*p* = 0.02). For female faces, the mean response to faces previously seen after placebo on day 1 was significantly lower in men given 20 IU on day 2 than in men given 40 IU on day 2 (−1.42, 95%CI: −2.65 to −0.15, *p* = 0.02). Again, we suspect this difference may reflect starting differences between the small numbers of men given 20 and 40 IU on day 2.

### Model Variations

None of the patterns in the models were affected by dropping four subjects with extreme emotional trauma (more than 2.5 SD from the mean for the average scores of emotional neglect and emotional abuse). Nor were they altered by dropping individuals who were not exclusively heterosexual, or adding RS3 allelic variation as a covariate (whether individuals had 0, 1, or 2 long alleles, and whether they had 0, 1, or 2 versions of the 335 allele). However, when we specifically compared responses to faces in men who had received 20 or 40 IU on day 1, the day when AVP effects were evident, between those who carried at least one 335 risk allele and those who did not, we found preliminary evidence that the allele may influence responsiveness to AVP. For single men, the mean Approachability rating of faces observed on day 1 was marginally lower in single men who received 20 IU and carried at least one copy of 335 (*n* = 6) than in men who received 20 IU but did not have a copy (*n* = 9; −1.02. 95%CI; −2.06 to −0.2, *p* = 0.05). For coupled men, the mean Initiate rating of female faces on day 1 was marginally higher in those who received 40 IU and did not carry a 335 copy (*n* = 6) than in men who received 40 IU but did carry at least one copy (*n* = 8; 0.92, 95%CI; −0.02 to 1.85, *p* = 0.05). Similarly, the mean Attractiveness rating of female faces observed on day 1 was marginally higher in coupled men who received 40 IU and did not carry a 335 copy (*n* = 6) than in those who received 40 IU and did carry at least one copy (*n* = 8; 1.13, 95%CI: 0.02 to 2.24, *p* = 0.05). Together, these preliminary findings are generally consistent with the hypothesis that carrying 335 increases acute, negative responses induced by low doses of AVP and decreases positive influences potentially induced by higher doses. However, we note that while there were dose differences associated with AVP delivery on day 1 in coupled men, we were unable to determine to what extent, if at all, those differences were associated with increased responses induced by 40 IU.

## Discussion

In this study two doses of intranasal AVP administration differentially influenced subjective responses to faces in men, and those influences differed between men who reported being single and those who reported being in a relationship. In single men, the lower dose, relative to the higher dose and, for Initiate, relative to placebo, generally decreased ratings of faces, although the most persistent differences were selective for male faces. On the other hand, lowered responses associated with 20 IU relative to 40 IU were more selectively associated with female faces in coupled men, and the dose differences toward men even reversed, with 20 IU associated with higher ratings than 40 IU for Attractiveness. Although we could not show that the higher dose increased positive ratings of the faces relative to placebo in this study, it did, several days after drug delivery, in a parallel fMRI study that measured some of the same behavioral responses [(44), see further discussion below]. Together, these studies suggest that different doses of AVP produce opposing effects on some social responses, perhaps as a function of different patterns of peptide receptor activation in the brain. Although the higher dose did appear to influence one peripheral response, it decreased, rather than increased, systolic BP, an effect inconsistent with peripheral vasoconstrictive influences and therefore suggestive of a central mechanism of action. Most importantly, and consistent with our parallel fMRI studies (44), some of the effects of intranasal AVP administration appeared potentially long lasting.

Similar dose differences were observed in single men for responses related to how approachable other faces appeared and how likely they would be to initiate conversation with those individuals. On the other hand, differences for Attractiveness, a potential index of sexual/romantic interest, were not different in single men given 20 and 40 IU, nor different from placebo for either dose. In contrast, the higher doses of AVP did increase Attractiveness ratings several days after drug adminstration in our parallel study in single, heterosexual men, though only to same-sex faces, suggesting that effect was also unrelated to sexual/romantic interest (44). All three behavioral responses were highly correlated and each likely reflects a global social assessment of the faces that the different populations of subjects in the two studies, primarily urban Caucasian men in this study and a diverse group of college students in our parallel study, may have emphasized/used differently. We are, therefore, hesitant to try to speculate on AVP influences on specific psychological parameters, but rather suggest that AVP manipulations generally affected tendencies to see others more or less positively, with lower doses promoting less positive assessments relative to high doses, and for Initiate, at least on day 1 in single men, relative to placebo. This pattern is consistent with the negative effects of 20 IU in our previous study (38). Unfortunately, we could not conclusively determine if 20 IU decreased, and/or 40 IU increased, ratings in the contrasts across test days, likely because of variation related to ratings of different individuals across test days and/or carry-over drug order effects that will be discussed below. However, it is worth noting that Approachability and Initiate ratings across the 2 days, and on the final day, remained lower in men given 20 IU than the “baseline” ratings of faces after placebo on day 1. We have observed that Approachability ratings of male faces generally increase across days in men because of experience with the faces and/or procedure [(44); Initiate was not measured in that study]. However, that did not happen in men given 20 IU in the current study, consistent with negative, lasting effects of the lower dose.

We were unable to conclusively demonstrate acute or lasting positive effects of 40 IU on ratings of male or female faces in the present study, but that dose did enhance Attractiveness ratings in our parallel fMRI study on a follow-up test days after drug delivery. The inability to detect similar effects of 40 IU in the present study may be the result of differences in experimental design and/or drug delivery methods in the two studies. The within-subjects nature of the current design may have obscured any such effects (see further discussion below), and the two studies used different drug delivery devices. We used a device that delivered the complete dose in a single, small volume of spray in an effort to avoid the leakage that we have observed sometimes accompanies repeated sniffs, which would decrease accuracy of the doses delivered. In our parallel fMRI study (44), in contrast, the dose was delivered *via* multiple sniffs. If effective entry into the brain depends on repeated sniffs and saturation of the nasal mucosa, as was suggested in a critical review of intranasal delivery methods (45), then higher central elevations may have occurred in subjects given 40 IU in the fMRI study than in subjects given 40 IU in this study. However, even with those delivery differences and associated difficulties comparing elevations of AVP likely induced within the central nervous system between the studies, together they suggest that different doses of intranasal AVP may produce opposing effects on some behaviors. We suggest lower doses decrease social assessments of others and higher doses increase them.

Arginine vasopressin and its non-mammalian homolog AVT dose-dependently affect social behaviors in other species. For at least some behaviors, there is an optimal dose associated with an inverted U dose relationship (8, 46, 47). This suggests that particular patterns of receptor activation have unique behavioral effects and that increasing doses produce patterns that counteract those induced by lower doses or produce different behavioral outcomes altogether. In this and our parallel study, the higher dose could have more broadly activated different types of receptors to which AVP has lower binding affinities, including OT receptors. OT can stimulate affiliative interactions in numerous species, including humans [reviewed in Ref. (48)], so increasing cross talk with such receptors at higher doses could negate antisocial effects of lower doses, which we propose happened in the present study, or produce positive responses, as observed in our parallel study. Dose-dependent receptor cross talk may not be purely pharmacological. Studies in rodents have not only shown that exogenous administration of AVP or OT can affect social behavior through promiscuous receptor activation (49, 50), but also that endogenous AVP and OT exert influences on some behaviors, including affiliative interactions, through receptor cross-talk mechanisms (18, 51).

On the other hand, it is possible that the effects of one or both doses are purely pharmacological. We do not yet know what the local concentrations of AVP in different brain circuits are following intranasal delivery of either dose or even what physiologically relevant concentrations of AVP are within individual circuits during social interactions in humans, though levels in extracellular space may be quite high, as they are in rodents (52). Furthermore, it is possible, if not probable, that intranasal peptide delivery simultaneously affects multiple circuits and induces patterns of brain activity, directly or as a result of feedback from the periphery, which could have happened in response to the higher dose in this study, which decreased systolic BP, that are not typical of any that occur in natural contexts. Those “unique” patterns could then produce behavioral influences that are not reflective of endogenous AVP functions. Thus, while this and other studies that have utilized intranasal methods do shed some light on potential roles that endogenous AVP systems play in social regulation, they should be interpreted cautiously. However, such studies do highlight the complexity and diversity of effects that pharmacologically targeting AVP systems may have upon behavior in clinical settings, some of which could be quite unintended.

Our results suggest some stimulus specificity for intranasal AVP’s influences on face processing, though they are complex, depending on duration (acute vs long term), relationship status, and possibly genotype. In single men, comparisons on day 1 indicated that while 20 IU, relative to 40 IU, generally decreased ratings of faces, the only significant difference between 20 IU and placebo was for Initiate responses to male faces. We also identified what appeared to be carry-over differences between single men given 20 and 40 IU on day 1 to new faces observed on day 2, though only toward new male faces. In our parallel study, long-term effects of AVP in single men were selective for male faces (44). Likewise, 20 IU AVP has been shown to selectively affect men’s ability to process emotional cues in the faces of other men, but not women (36). Together, these studies indicate that AVP plays a predominant role in the processing of same-sex faces in men, but that its effects are not exclusive to males face processing. Indeed, in coupled men at least some of AVP’s influences appeared more selective for female faces, the ratings of which, at least for Attractiveness, were lower in men given 20 IU than in men given 40 IU.

It is possible the differences in stimulus specificity for AVP influences in single and coupled men were related to different perceptions of the social context of the rating task. The juxtaposed presentation of male and female faces, rated on attributes associated with interpersonal interactions, including those potentially related to sexual interest, could have created a context of reproductive competition that was perceived differently in single and coupled men. In single men, the male faces may have represented a source of threat/competition for the female pictured, and thus the most persistent influences of AVP in single men, which we argue reflect decreased ratings associated with 20 IU, were antisocial responses toward other male faces. Of course, as mentioned, not all of the dose differences were exclusive for male faces, suggesting that at least some of AVP’s influences may be part of a more generalized response that decreases assessments of faces, perhaps in relation to AVP’s ability to increase stress responses in conditions of social threat (53). It is also possible that some of the dose differences reflect, in part, increased ratings of faces, particularly male faces, induced by the higher dose. If so, that would be consistent with positive effects induced by 40 IU in our parallel study, albeit on a slower time scale (44).

In men who reported being in a romantic relationship, on the other hand, in which the lower dose, relative to the higher dose, was selectively associated with decreased ratings of female faces, the perceived social context may have been different. It is possible that the novel female was the larger perceived threat to those men, who were rating the attractiveness of unfamiliar women in the absence of their partner. Thus, 20 IU may have lowered social assessments of this threat to their current relationship. Similarly, intranasal OT selectively promotes withdrawal from unfamiliar women in men who are in relationships (54). The faces of other men, on the other hand, would presumably not be a rival for the absent partner. In those contexts, the dose differences even appeared to change, with the lower dose increasing ratings of the male faces relative to the higher dose. It is also possible the dose difference in responses to female faces reflect, in part, increased responses induced by 40 IU. Although 40 IU did not increase ratings of female faces in single men in our parallel study, it did selectively increase neural responds in the ventral striatum and septum to female faces (44), both areas in which nonapeptides induce affiliative responses related to pair bonding in prairie voles (see further discussion below).

This context-dependent explanation for potential differences in stimulus specificity between single and coupled men would suggest that AVP has a common effect on the brains of single and coupled men, and that the divergent behavioral outcomes of that effect are a function of differences in perceived social contexts. Alternatively, it is possible that AVP differentially affects the brains of single and coupled men. Pairing can influence vasopressin receptor expression and change responsiveness to social stimuli in prairie voles (17, 55), so such a mechanism is possible. In our parallel study, we only measured the effects of 40 IU in single men on brain responses and not of the lower dose that we suggest decreased social assessments in this and our previous study (38). It will, therefore, be interesting to determine if AVP produces different patterns of brain responses to female and male faces in single and coupled men, particularly in nodes of the Social Brain Network, or if it induces similar patterns across those subject populations. If the latter occurs, it would suggest that behavioral differences between single and coupled men are a downstream consequence of that common activation, filtered by social context.

We did not detect specific effects of AVP relative to placebo for either dose in our within-subjects comparisons across days, and many of the dose differences were qualified by drug order. Those interactions were largely associated with AVP delivery on the first test day. Responses to faces on the first day were lower in single men given 20 than 40 IU, as were responses to the faces seen after placebo on the second test day, and responses to female faces were lower in coupled men given 20 IU relative to 40 IU on the first day. Additionally, responses on the final day to the faces previously seen across the first 2 days were lower in men who had received 20 IU on day 1 than in men who had received 40 IU. These patterns suggest that acute AVP effects may be most pronounced in novel/ambiguous test contexts (the first day), and that some of its effects may be long lasting and potentially generalizable to faces seen subsequent to AVP’s initial administration. We had originally predicted that AVP would acutely affect responses to faces independent of the day of drug delivery, and that any lasting influences would be selective for the faces paired with drug. We did find some evidence for more selective, long-term effects of AVP for faces seen immediately after AVP in our parallel study (44). In that study 40 IU AVP increased positive ratings of the male faces paired with AVP 2–21 days after AVP delivery, but not of a novel face seen for the first time on that final test day. It remains to be resolved whether differences in how selective or generalized the lasting influences of AVP were in the two studies reflect unique mechanisms induced by the different doses in relation to their promotion of positive [high dose (44)] and negative (low dose, current study) responses or simple differences in study design.

The mechanisms through which either dose of intranasal AVP may produce prolonged negative and/or positive effects on face evaluations are entirely unclear. Acute elevations of AVP enhance social learning and memory processes in rodents, presumably by altering connectivity within neural networks (24, 56–58). However, those influences are selective for specific individuals encountered immediately before AVP administration; in the current study, at least, AVP effects appeared to influence responses to faces seen after drug delivery and to generalize to new faces seen on subsequent days, which suggest an alternative mechanism that could involve lasting, general influences on social stimulus processing. It seems unlikely that a single dose of AVP could induce epigenetic changes within those circuits, yet it does remain a possibility, especially if intranasal AVP can trigger feed-forward mechanisms that facilitate further and possibly prolonged release (59). Recent studies have demonstrated epigenetic modifications induced by the cohabitation/mating experiences that trigger AVP release and, as a result, induce pair bonding in prairie voles (25, 26), but it is not yet known if the AVP released during those interactions contributes to the epigenetic modifications. It is also possible the prolonged behavioral influences in this and our parallel study do not reflect lasting effects of acute elevations on the brain, which is easy to presume because of the short half-life of AVP in tissue (60). Rather, they could be a function of lasting elevations of AVP induced by the intranasal administration. Despite its short half-life in tissue, levels of AVP in cerebrospinal fluid were still elevated 120 min after drug delivery in the original studies by Born et al. (28). As mentioned, AVP has been shown to facilitate feed-forward mechanisms that can promote further release within the brain (59), raising the possibility of a surprisingly long-window in which intranasally delivered AVP could influence social/emotional processes.

AVP did not promote positive ratings of female faces, acutely or over time, even when we restricted the model to heterosexual, single men. We had hypothesized that AVP might, in single men as in unpaired male prairie voles, stimulate affiliative processes that would promote interactions with potential reproductive/romantic partners, manifested as more positive ratings of female faces. Our parallel fMRI studies also failed to detect AVP effects on behavioral responses to female faces in single men, though 40 IU AVP did selectively increase activation in the ventral striatum and septum, areas in which AVP and OT modulation influences affiliative behaviors related to pair bonding in prairie voles, when males looked at female faces. Thus, it is possible that our behavioral measures simply did not capture responses related to tendencies to form emotional attachments in reproductive contexts. It should also be noted that continuous infusions of AVP, while males have extended contact with females, are required to enhance partner preferences in male prairie voles; single injections are not effective (18). We only delivered AVP once, and interactions with females were limited to brief exposures to their faces, which were devoid of positive emotional cues that are likely necessary to promote affiliative interactions. Thus, it is likely these tests did not promote the concomitant dopamine release that normally occurs during cohabitation in voles and that is necessary for AVP to induce partner preferences (61). Of course, life histories associated with pair bond formation in reproductive contexts evolved independently in most lineages in which such behaviors are evident, including humans, and there is not yet conclusive evidence for convergent AVP/AVT mechanisms that promote pair bonding in males across those species (62–64). Thus, it is also possible that endogenous AVP does not play a role in pair bond formation in reproductive contexts in humans, or that it plays a role in relationship maintenance, rather than pair bond formation. In another primate that forms long-term pair bonds, titi monkeys, intranasal AVP increases social contact with already established mates in males (65).

We also ran models that included RS3 allelic variation previously associated with differences in social responses (27, 66–71). Inclusion as a covariate of whether men had one or two long alleles (≥335), or one or two copies of a previously identified risk allele (335 with our primers), did not influence the pattern of results. However, our preliminary, focused analysis of whether having at least one copy of the 335 risk allele in men given AVP on the first test day, when the drug appeared most likely to influence behavior, suggest the allele may, as predicted, be associated with more negative, antisocial responses to AVP. In single men, Approachability responses were lower in men given 20 IU on the first day if they carried at least one copy of 335, and Attractiveness responses were not as high in coupled men given 40 IU who carried at least one copy as in those who did not. These patterns suggest the risk allele may increase negative social assessments associated with low doses and/or decrease positive assessments associated with higher doses. Consistent with the possibility that AVP might induce more negative/less positive social assessments in carriers of the risk allele, men with that allele show heightened amygdala responses to faces (43). Unfortunately, we did not have sample sizes sufficient to evaluate more fully the relationship between V1a gene variation and responsiveness to AVP.

### Study Limitations

There are several limitations with the current study and with intranasal delivery studies more generally, some of which have already been discussed. Intranasal delivery is likely highly variable as a simple function of individual competence with self-delivery. Furthermore, we know nothing about local elevations in individual brain regions that follow the delivery of different doses, or even about elevations within cerebral spinal fluid that result from the same dose delivered with different applicators. We agree with Churchland and Winkielman’s (47) argument that a systematic study comparing elevations in CSF using different applicators would be quite helpful in that regard, as would determining how long the elevations persist. More specifically related to this study, the stimuli used were all Caucasian, and the study population was largely limited to Caucasians. Therefore, we suggest caution about generalizing results related to dose-dependencies or long-term influences to non-Caucasian populations or to influences on responses toward more diverse groups of subjects that could be moderated by in-group/out-group perceptions. Perhaps most importantly, the unexpected carry-over/lasting influences of AVP discovered in this and our parallel study suggest that within-subjects, repeated measures designs, even when drug order is counterbalanced and statistically accounted for, are difficult to interpret. Such designs are common in non-human studies of peptide effects, under the presumption that these peptides do not produce long-term influences on behavior in adults. We suggest future studies should consider potential long-term and drug-order effects more carefully.

## Conclusion

The present results suggest that AVP produces dose-dependent influences on face processing in men, that those influences differ as a function of relationship status, and that some may be long lasting and potentially generalizable to faces seen after the initial drug delivery. The potential for intranasal AVP to induce long-lasting effects on behavior, in particular, warrants further discussion on the use of this method for basic research and the implications that might be associated with clinical interventions that pharmacologically target the AVP system.

## Ethics Statement

All subjects received informed consent, and the protocol was approved by the Bowdoin and Maine Medical Institutional Review Boards, as well as by the US Food and Drug Administration.

## Author Contributions

DP participated in study design, evaluated all subjects, oversaw day-to-day running of the experiment, and wrote the paper. DB and AC ran all subjects, kept all logs, and advised on technical aspects of study implementation. CT ran all statistics and wrote the paper. JR participated in study design and wrote the paper. RT participated in study design, data interpretation, and wrote the paper.

## Conflict of Interest Statement

The authors declare that the research was conducted in the absence of any commercial or financial relationships that could be construed as a potential conflict of interest.
