# Corrigendum: Dose-Dependent and Lasting Influences of Intranasal Vasopressin on Face Processing in Men

**DOI:** 10.3389/fendo.2017.00334

**Published:** 2017-11-28

**Authors:** Daniel Price, Debra Burris, Anna Cloutier, Carol B. Thompson, James K. Rilling, Richmond R. Thompson

**Affiliations:** ^1^Department of Psychiatry, Maine Medical Center, Maine Health, Portland, OR, United States; ^2^Biostatistics Center, Bloomberg School of Public Health, Johns Hopkins, Baltimore, MD, United States; ^3^Department of Anthropology, Emory University, Atlanta, GA, United States; ^4^Department of Psychiatry and Behavioral Science, Emory University, Atlanta, GA, United States; ^5^Center for Translational Social Neuroscience, Emory University, Atlanta, GA, United States; ^6^The Center for Social Neuroscience, Atlanta, GA, United States; ^7^Neuroscience Program, Psychology Department, Bowdoin College, Brunswick, GA, United States

**Keywords:** social behavior, V1a receptor, social context, intranasal, face processing

In the original article, there was a mistake in the legend for Figure 5 as published. The legend should have only alluded to one dotted line in each panel indicating average responses to all faces on day 1 in single men who received placebo on that day. The correct legend appears below.

**Figure 5:**
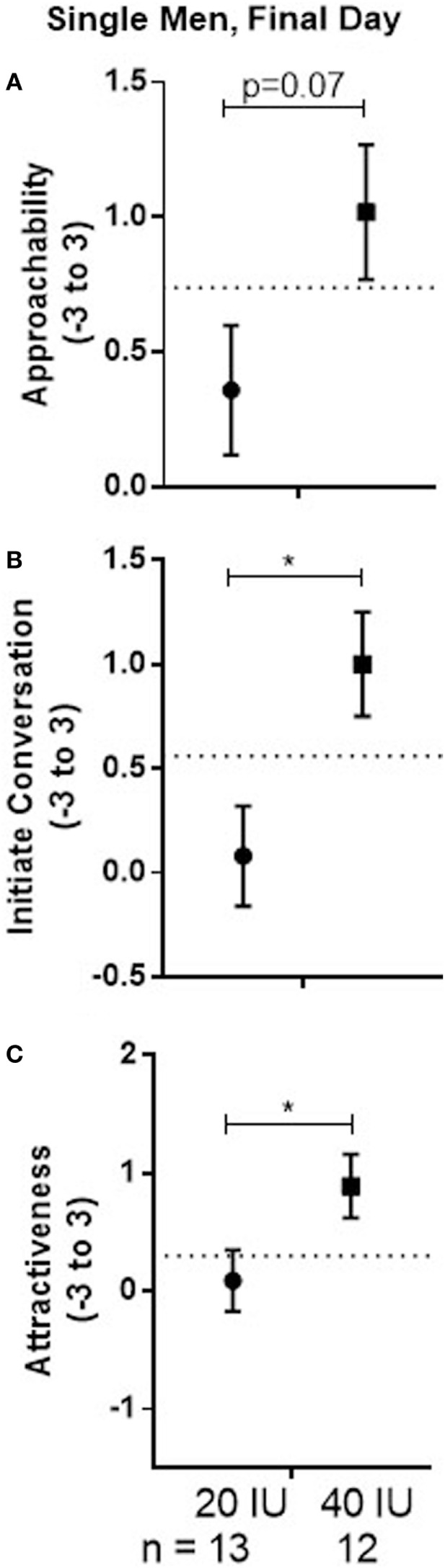
Mean ± SEM of approachability **(A)**, initiate **(B)**, and attractiveness **(C)** ratings, averaged across sex, on the final day of testing when no drug was given in men who received 20 or 40 IU on day 1. The dotted line shows mean response to all faces on day 1 in men who received placebo on that day.

In the original article, there was a mistake in Figure 5 as published. The subject numbers at the bottom of the graph were incorrect. The corrected Figure 5 appears below.

The authors apologize for these errors and state that these do not change the scientific conclusions of the article in any way. The original article was updated.

## Conflict of Interest Statement

The authors declare that the research was conducted in the absence of any commercial or financial relationships that could be construed as a potential conflict of interest.

